# A Survey to Evaluate the Association of COVID-19 Restrictions on Perceived Mood and Coping in Australian Community Level Athletes

**DOI:** 10.3389/fspor.2021.624267

**Published:** 2021-03-22

**Authors:** Caitlin Fox-Harding, Sarah Ann Harris, Shane L. Rogers, Shayne Vial, Philipp Beranek, Mitchell Turner, Travis Cruickshank

**Affiliations:** ^1^School of Medical and Health Sciences, Edith Cowan University, Perth, WA, Australia; ^2^Institute for Health Research, The University of Notre Dame Australia, Fremantle, WA, Australia; ^3^Centre for Precision Health, ECU Strategic Research Centre, Edith Cowan University, Perth, WA, Australia; ^4^Perron Institute, QE II Medical Centre, Perth, WA, Australia

**Keywords:** 2019-nCoV (2019 novel coronavirus), Australia, athlete, survey, coping

## Abstract

**Highlights:**

- No differences in coping or well-being in Australian community athletes based on the level of support received during COVID-19 restrictions.
- Community level athletes had better coping when a training program was provided.
- No difference between individual or team community athletes for well-being or coping scores.

Australian community level athletes faced unprecedented changes to their training and competition options as the global COVID-19 pandemic took a stronghold. This disruption was predicted to have a negative impact on emotional well-being as communities braced through periods of social isolation and physical distancing requirements. This study provides an Australian perspective on the emotional well-being of community level athletes and the extent to which they coped during the COVID-19 pandemic. Emotional well-being and coping were measured using the Brief Emotional Experience Scale and the 28-item Brief Cope Scale. Both instruments were administered along with other questions pertaining to participant demographics and training status via an online survey between April and June 2020. The survey was disseminated to community athletes through word-of-mouth and social media platforms. No significant differences in emotional well-being were observed between athlete groups as a result of COVID-19 and its associated restrictions. Coping scores also appeared to be preserved in Australian community athletes, which contrasts the impact expected as a result of the COVID-19 pandemic. While tentative, the observed preservation in coping may have buffered potential declines in emotional well-being, which has been documented in professional and semi-professional athletes and the general population. These unexpected findings and tentative suppositions warrant further investigation and highlight the importance of conducting a country- or region-specific approach to examining the impact of COVID-19 on community athletes, as responses to COVID-19 are undoubtedly not consistent throughout the world.

## Introduction

COVID-19 has caused significant emotional distress worldwide (Montemurro, 2020; Pfefferbaum and North, 2020; Shanahan et al., 2020). Fear of infection (Lazzerini et al., 2020) and implementation of preventative health measures, including social and physical distancing (Galea et al., 2020) by governments to reduce COVID-19 transmission has seen a dramatic increase in global mood disorders (Kumari and Mahla, 2020; Van Rheenen et al., 2020). Populous level studies conducted in Italy, Spain, USA, and Australia have noted a significant increase in stress, anxiety and depressive symptomology (Fitzpatrick et al., 2020; Ozamiz-Etxebarria et al., 2020; Rossi et al., 2020; Staples et al., 2020). Together, these studies highlight the significant impact of COVID-19 on the general population, however, provide little insight into the consequences of this pandemic on specific societal populations, including the sporting community.

Many studies examining the sporting community have investigated the impact of COVID-19 on adolescent mental health (McGuine et al., 2020a,b), large sporting events (Adami et al., 2020; Mann et al., 2020), as well as professional and semi-professional athletes (Hughes et al., 2020; Ludvigsen and Hayton, 2020; Murray et al., 2020). These studies have noted elevated anxiety, depression, and greater perceived stress in South African (Pillay et al., 2020) and Italian athletes (di Fronso et al., 2020), respectively. These adverse changes in mood state were attributed to a disruption to sporting events (e.g., cancellation of competitions), which negatively impacts professional standing (e.g., ranking) and salary (e.g., loss of income). While important, it is noteworthy that semi-professional and professional athletes only represent a small percentage of the sporting community, with community athletes representing a significantly larger population (May, 2020). Although research has confirmed that community level athletes account for 90% of the entire Australian athletic population per the Clearinghouse for Sport 2019 data, there is a paucity of research which has specifically examined the impact of COVID-19 on community level Australian athletes.

In one of the few available studies, di Fronso et al. (2020) explored the impact of COVID-19 on perceived stress and emotional well-being in professional and community level Italian athletes. Greater stress and poorer emotional well-being were noted in community level athletes when compared to elite/expert athletes, which authors attributed to a reduced ability to deal with adversity and effectively regulate emotions (Anshel and Kaissidis, 1997; Anshel et al., 2009). Makarowski et al. (2020) reported no changes in perceived stress in a large sample of athletes from eastern and western Europe as well as parts of Asia. Authors attributed this unexpected finding to greater utilization of avoidant (i.e., maladaptive) coping strategies, including denial, substance use and venting. Although a form of coping, maladaptive strategies will not facilitate long-term relief from the perceived difficult situation (Brown et al., 2005; Cheng et al., 2020; Rettie and Daniels, 2020). As such, the type of coping strategy being utilized is an important differentiating factor to encourage long term coping relief. Together, these findings suggest that COVID-19 differentially impacts the mood state of community athletes, which may be explained, at least in part, by employed coping strategies, sport and country of residence. The preliminary nature of these findings necessitates further investigation and comparison throughout other countries.

The purpose of this study was to examine emotional well-being and coping strategy utilization in Australian community level athletes during the COVID-19 pandemic. It was hypothesized that emotional well-being would be negatively impacted when a lower level of support was received during the COVID-19 pandemic and associated with a diminished coping capacity in Australian community level athletes. Exploring potential effect on Australian community level athletes provides a unique insight into the importance of community level sport.

## Materials and Methods

### Ethics Approval

This study was approved by the Edith Cowan University Human Resource Ethics Committee (HREC: 2020-01315).

### Study Design/Participants/Setting

This is a cross-sectional descriptive study. The questionnaire was electronically distributed throughout Australia during April-June 2020 via word-of-mouth and social media platforms including Twitter, Facebook and LinkedIn. Participants were required to compete at a community level, have engaged or intended to engage in a formalized sporting competition or training prior to the COVID-19 restrictions (March 2020), reside in Australia and be aged 18 years and over to participate. Due to the unprecedented and unpredictable nature of COVID-19, a sample size estimation was not completed.

For the purpose of this study, “lock down” was defined as the closure of Australian international and interstate borders. All Australian states and territories allowed individuals to exercise outdoors but recommended they stay home unless absolutely necessary. Restrictions varied from state to state, organized sport ceased, and gym facilities were indefinitely closed with the eastern seaboard being affected the most. Australian athletes were able to continue training throughout the nation with modified (e.g., home-based) activity and maintain a sense of connectedness using electronic means.

### Measures

An online survey was administered using Qualtrics software (Appendix 1). Within the survey, participants were asked to report on their current demographics (nine-items), COVID-19 sporting relationship (20-items), perceived emotional well-being (six-items), and coping (28-items).

Coping was assessed using the 28-item Brief COPE Scale (Carver, 1997). Items were scored using a four-point Likert Scale, 1 (I haven't been doing this at all) to 4 (I've been doing this a lot). Possible total scores ranged from 28 to 112 with a lower score indicating poorer coping strategies. In addition, 12 of the 14 subscale scores can be grouped to reflect two key domains of Avoidant Coping (sum of 12-items, items: 1, 3, 4, 6, 8, 9, 11, 13, 16, 19, 21, 26) or Adaptive Coping (sum of 12-items, items: 2, 5, 7, 10, 12, 14, 15, 17, 20, 23, 24, 25) (Carver, 1997; Mahmoud et al., 2012).

Perceived emotional well-being was assessed using the Brief Emotional Experience Scale (Rogers et al., 2016; Skead and Rogers, 2016; Skead et al., 2018, 2020; Rogers and Cruickshank, 2020; BEES). Participants reflected on how they have felt emotionally over the previous month across six-domains (three positive—happy, calm, confident; and three negative—worried, safe, afraid). The six emotional items were scored using a four-point Likert Scale, one (Not at all), two (a little bit), three (quite a bit), four (a lot or extremely). To calculate the total score, the mean of three negative items is subtracted from the mean of three positive items. Total possible score ranged from −3 to +3, with a higher score indicating “higher emotional well-being.” Additionally, a cut off score of >0 indicates “positive emotion is greater than negative emotion.”

Both scales are self-reported by the participant and have established validity and reliability within the general population (Carver, 1997; Rogers and Cruickshank, 2020).

### Data Analysis

Descriptive statistics [Sample Size (N), Mean (M), Standard Deviation (SD), Median (Mdn)] for emotional well-being (BEES) and coping (Coping) scores were calculated based on participant demographics (e.g., Age, State) and sporting details (e.g., team or individual). Individual univariate generalized linear models explored the association between each outcome variable (BEES, Total Coping, Avoidant Coping, Adaptive Coping) and each independent variable (demographic and sporting variables). Sub-categories were excluded if the cell count was <3.

Four generalized linear models explored if the level of support provided to the athlete was associated with a community level athlete's ability to cope during COVID-19, while adjusting for other extraneous factors. If any sub-categories of the five key independent variables of interest (Club Support) had a cell count of <3, they were excluded from all four models. Details of each model set up within SPSS included: A generalized linear model, with a linear scale response (with identity link function). Club support, demographic and sport-related variables (factors) were assessed with category order per factor specified in descending order. Model effects were specified as main effects and Type III model effect, with the intercept included in the model and 95% confidence intervals The scale parameter was set to Maximum likelihood estimate and the log-likelihood function set to full, with model based exponential parameter estimates included. Standardized residuals were saved for visual assessment.

Ability to cope was explored across four separate models (one dependent variable per model) including emotional well-being (Total BEES score), Total Coping (sum score), Avoidant Coping (sum score), and Adaptive Coping (sum score). Club support was explored by five independent variables. These variables included contact by the national sporting organization (No, Yes), contact by the state sporting organization (No, Yes), contact by the club-level sporting organization (No, Yes), ability to continue training (No, Yes), and provided with a training program (No, Yes). Each model was also adjusted for five demographics factors—state or territory, sex, age, change in employment due to COVID-19, and medical condition. Additionally, the models were adjusted for two sport-related fixed factors including team or individual sport and position security within the sport. For each model (1–4), residuals were visually inspected for normality.

Data analysis was completed in Microsoft Excel and IBM SPSS Statistics Version 25. Significance was set at p < 0.05 for all analyses. There was no missing data due to the parameters of the Qualtrics study set to require all survey items to be completed.

## Results

### Participants

A total of 151 participants completed the questionnaire responding across all eight states or territories of Australia, with Western Australia accounting for 64% of responses (Table 1). There was no significant difference in emotional well-being (BEES), Total Coping, Avoidant Coping or Adaptive Coping between states or territories. While not significant, participants from Northern Territory and Tasmania had the greatest coping scores (although the lowest respondent rate, *n* = 2), with participants from New South Wales and Victoria displaying lower coping scores. The majority of participants identified as female (*N* = 89, 59%) and were in a relationship (*N* = 111, 74%). Initial univariate analysis indicated a significant difference in emotional well-being (BEES) for sex (*p* = 0.003), with females reporting lower mean scores (M = 0.30, SD = 1.27) compared to males (M = 0.92, SD = 0.92) (i.e., females had a lower emotional well-being than males). Total coping scores were significantly greater in females (M = 57.16, SD = 9.97, *p* = 0.026), in comparison to males (M = 53.33, SD = 10.85). There was a significant difference in Adaptive Coping scores for sex (*p* = 0.019), with females reporting higher mean scores (M = 30.15, SD = 6.87) compared to males (M = 27.48, SD = 6.87) (i.e., females had greater adaptive coping than males). There was no significant difference in Avoidant Coping scores based on sex (*p* = 0.082), although descriptively females had a greater mean Avoidant Coping score (M = 20.02, SD = 4.30) than males (M = 18.73, SD = 4.72). A fifth of participants were aged between 26 and 30 years (*N* = 30, 20%), whilst the age ranged from 18 years to over 71 years. Avoidant coping scores were significantly greater (*p* = 0.009) for 18–21 years (*p* = 0.008), 22–25 years (*p* = 0.003), 26–30 years (*p* = 0.025), 31–35 years (*p* = 0.017), 36–40 (*p* = 0.029), and 51–60 (*p* = 0.016) in comparison to participants aged 71 years and over. Although not significant, older respondents had greater emotional well-being scores (*p* = 0.144) and lower total coping scores (*p* = 0.376) (poorer ability to cope) than younger participants.

**Table 1:** Participant demographics (*N* = 151).

	***N***	**%**	**BEES**			**Coping**			**Coping avoidant**			**Coping adaptive**		
			**M**	**SD**	**Mdn**	**M**	**SD**	**Mdn**	**M**	**SD**	**Mdn**	**M**	**SD**	**Mdn**
**Total responses**	151	100.00	0.58	1.32	0.67	55.65	10.43	56.00	19.48	4.48	19.00	29.13	6.96	29.00
**Location**														
ACT	4	2.65	0.67	2.16	1.17	52.75	4.99	52.50	18.75	4.50	17.50	27.75	3.59	26.50
NSW	15	9.93	1.33	1.08	1.33	51.33	11.65	48.00	17.13	3.96	16.00	26.67	6.87	28.00
NT ^b^	2	1.32	−0.33	0.94	−0.33	68.50	3.54	68.50	21.00	2.83	21.00	39.50	0.71	39.50
QLD	10	6.62	0.30	1.54	0.67	59.30	12.18	60.50	20.20	4.42	20.50	31.30	8.30	33.50
SA	5	3.31	0.87	1.04	0.33	59.00	4.95	59.00	22.40	5.55	20.00	29.60	3.29	29.00
TAS ^b^	2	1.32	−0.33	2.36	−0.33	62.00	14.14	62.00	24.00	8.49	24.00	31.50	2.12	31.50
VIC	17	11.26	0.63	1.26	0.33	53.12	9.12	52.00	18.82	3.28	19.00	28.12	6.94	25.00
WA	96	63.58	0.51	1.29	0.67	55.94	10.44	56.50	19.64	4.58	19.00	29.24	7.06	29.00
**Sex**														
Male	60	39.74	0.92	1.26	0.67	53.33	10.85	52.50	18.73	4.72	17.50	27.48	6.85	28.00
Female	89	58.94	**0.30**^a^	1.27	0.33	**57.16**^a^	9.97	58.00	20.02	4.30	19.00	**30.15**^a^	6.87	30.00
Other/ Prefer not to say^b^	2	1.32	3.00	0.00	3.00	58.00	9.90	58.00	17.50	2.12	17.50	33.50	6.36	33.50
**Age**														
18–21 years	17	11.26	0.51	1.02	0.67	56.76	12.32	56.00	**20.59**^a^	4.96	19.00	28.94	6.91	28.00
22–25 years	24	15.89	0.14	1.18	0.17	58.96	9.92	58.50	**21.42**^a^	4.80	20.00	30.08	6.16	29.00
26–30 years	30	19.87	0.54	1.37	0.67	56.40	8.78	57.50	**19.37**^a^	3.90	19.00	29.93	6.47	29.50
31–35 years	13	8.61	0.41	1.56	0.33	55.77	11.71	56.00	**20.08**^a^	3.30	20.00	28.00	8.76	26.00
36–40 years	9	5.96	0.67	1.37	1.00	53.89	8.74	54.00	**19.78**^a^	4.82	19.00	26.89	6.81	28.00
41–50 years	24	15.89	0.89	1.34	1.00	53.46	11.15	53.50	17.67	3.86	17.00	28.92	7.41	29.50
51–60 years	24	15.89	0.61	1.34	0.50	56.04	10.97	57.50	**19.83**^a^	4.81	19.50	29.33	7.72	27.50
61–70 years	7	4.64	0.71	1.28	0.33	50.43	9.54	49.00	16.57	3.82	16.00	28.86	7.15	30.00
71 years +	3	1.99	2.56	0.19	2.67	46.67	6.66	45.00	13.67	2.08	13.00	27.00	5.57	26.00

*M, Mean; SD, Standard Deviation; Mdn, Median*.

*The reference categories are set to Location (WA); Sex (Male); Age (71 Years +)*.

*BEES: Brief Emotional Experience Scale. Lower score indicates lower emotional well–being. Possible score −3 to +3*.

*Coping: Brief COPE Scale. Lower score indicates a poorer ability to cope. Possible score 28–112*.

*Coping Avoidant: Brief COPE Domain Scale. Higher score indicates stronger Avoidant coping traits*.

*Coping Adaptive: Brief COPE Domain Scale. Higher score indicates stronger Adaptive coping traits*.

a *Significant difference to reference category at p < 0.05*.

b *Sub-category excluded from analysis due to low cell count*.

*Bolded is to emphasize the “a” which denotes: Significant difference to reference category at p < 0.05*.

### Employment and Medical Conditions

During the study period, no participants were diagnosed with COVID-19 while two participants stated they were unsure (Table 2). Almost a fifth of participants lost their employment due to COVID-19 (19%), with an additional fifth of participants experiencing a reduction in employment (20%). Of the 30 participants with reduced employment, 61% (*N* = 17) saw their employment decrease by at least 50%.

**Table 2:** Comparison of emotional well-being and coping scores based on loss of employment due to COVID-19 and pre-existing medical conditions (*N* = 151).

	***N***	**%**	**BEES**			**Coping**			**Coping avoidant**			**Coping adaptive**		
			**M**	**SD**	**Mdn**	**M**	**SD**	**Mdn**	**M**	**SD**	**Mdn**	**M**	**SD**	**Mdn**
**Total responses**	151	100.00	0.58	1.32	0.67	55.65	10.43	56.00	19.48	4.48	19.00	29.13	6.96	29.00
**Employment loss due to COVID-19**														
Yes	28	18.54	0.28	1.32	0.17	58.73	10.35	59.50	20.40	4.41	19.50	30.87	6.65	31.50
Reduced employment	30	19.87	0.40	1.38	0.50	55.36	8.67	54.00	19.50	3.93	18.00	28.96	5.77	28.00
No effect	93	61.59	0.74	1.28	0.67	54.74	10.85	55.00	19.17	4.66	19.00	28.62	7.35	29.00
**Medical condition**														
None	115	76.16	0.66	1.23	0.67	55.28	10.69	55.00	19.52	4.58	19.00	28.83	6.84	29.00
Immune System ^b^	1	0.66	2.33	0.00	2.33	61.00	0.00	61.00	16.00	0.00	16.00	38.00	0.00	38.00
Immune System and Other ^b^	2	1.32	−0.83	0.71	−0.83	59.50	0.71	59.50	24.00	1.41	24.00	29.00	1.41	29.00
Respiratory	10	6.62	0.17	1.60	0.17	54.80	11.37	53.50	19.30	4.72	18.00	27.50	7.86	25.50
Respiratory and Other	3	1.99	−0.89	0.51	−1.00	63.00	7.55	62.00	24.67	0.58	25.00	31.00	7.21	29.00
Immune and Respiratory ^b^	0	0.00	-	-	-	-	-	-	-	-	-	-	-	-
Other	20	13.25	0.62	1.58	0.33	56.45	9.65	55.00	18.25	3.63	18.50	30.95	7.54	29.50

*M, Mean; SD, Standard Deviation; Mdn, Median*.

The reference category is set to “no effect” or “none.”

*BEES, Brief Emotional Experience Scale. Lower score indicates lower emotional well-being. Possible score −3 to +3*.

*Coping: Brief COPE Scale. Lower score indicates a poorer ability to cope. Possible score 28–112*.

*Coping avoidant: Brief COPE Domain Scale. Higher score indicates stronger Avoidant coping traits—Non-parametric alternative used*.

*Coping adaptive: Brief COPE Domain Scale. Higher score indicates stronger Adaptive coping traits*.

a *Significant difference to reference category at p < 0.05*.

b *Sub-category excluded from analysis due to low cell count*.

The mean coping score was greater for participants who lost all employment (i.e., coping better), than participant's whose employment was not affected. Comparatively, emotional well-being was greater in participants whose employment was not affected, in comparison to those who partially or completely lost employment (Table 2).

Emotional well-being scores appeared poorer for participants with respiratory and other illnesses compared to other medical conditions, or no medical conditions (Table 2). By contrast, coping appeared similar across all medical illnesses. Respondents with immune related medical conditions had the lowest avoidant coping scores, while participants with respiratory or immune and other medical conditions reported lower adaptive coping domains. Employment and Medical Conditions were not significantly associated with BEES, Coping, or Adaptive and Avoidant Coping.

### Sporting Profile and Effect of COVID-19

Participants responded across a plethora of team and individual sports (Supplementary Table 1), including Triathlon, Ten Pin Bowling, Running, Hockey, Fencing, Lawn Bowls, Cycling, and Australian Football. Three quarters of the participants were classified as participating in an individual sport (Table 3). Comparing team and individual athletes, team athletes had marginally greater emotional well-being scores, while individual athletes had slightly greater mean total coping score and adaptive coping scores. However, emotional wellbeing (*p* = 0.173), total coping (*p* = 0.135), avoidant coping (*p* = 0.108) and adaptive coping (*p* = 0.412) scores did not differ between sporting type (team or individual).

**Table 3:** Participant sporting details cross referenced with BEES, Coping and Coping Domain scores (*N* = 151).

	***N***	**%**	**BEES**			**Coping**			**Coping avoidant**			**Coping adaptive**		
			**M**	**SD**	**Mdn**	**M**	**SD**	**Mdn**	**M**	**SD**	**Mdn**	**M**	**SD**	**Mdn**
**Total Responses**	151	100.00	0.58	1.32	0.67	55.65	10.43	56.00	19.48	4.48	19.00	29.13	6.96	29.00
Type of sport^*^														
Team	113	74.83	0.68	1.31	0.67	54.82	10.39	55.00	19.08	4.26	19.00	28.84	7.11	29.00
Individual	37	24.50	0.35	1.27	0.33	57.73	10.17	57.00	20.41	4.76	20.00	29.92	6.55	29.00
Other^b^	1	0.66	−2.00	–	−2.00	72.00	-	72.00	30.00	–	30.00	33.00	-	33.00
**Eligible to play in 2020**														
Yes	141	93.38	0.58	1.31	0.67	55.32	10.44	56.00	19.33	4.39	19.00	28.97	7.05	29.00
No	10	6.62	0.67	1.45	0.33	60.30	9.73	65.50	21.60	5.46	21.00	31.40	5.30	29.00
Position in team^*^														
Deteriorated a lot	29	19.21	0.43	1.07	0.33	53.86	12.17	50.00	19.00	4.46	19.00	28.24	8.12	28.00
Deteriorated somewhat	30	19.87	0.42	1.28	0.67	56.00	9.57	55.50	20.53	4.61	19.50	28.60	7.43	28.00
No change	88	58.28	0.74	1.39	0.67	55.95	10.21	56.00	19.15	4.44	18.00	29.53	6.51	29.00
Improved somewhat	4	2.65	−0.42	1.26	−0.83	59.25	9.91	56.00	22.25	3.86	22.50	30.75	4.99	29.00
Improved a lot^b^	0	0.00	–	-	-	-	-	-	-	-	-	-	-	-
**Contact from sports organization**														
National level														
Yes	107	70.86	**0.42**^a^	1.24	0.33	55.73	10.54	56.00	19.46	4.44	19.00	29.18	6.78	29.00
No	44	29.14	0.99	1.42	1.00	55.45	10.29	56.00	19.52	4.62	19.00	29.02	7.45	29.00
State level														
Yes	107	70.86	0.53	1.23	0.67	56.06	10.63	56.00	19.50	4.48	19.00	29.38	7.00	29.00
No	44	29.14	0.71	1.51	0.83	54.66	9.99	54.50	19.41	4.55	19.00	28.52	6.90	28.00
Club level														
Yes	115	76.16	0.58	1.28	0.67	56.17	10.86	57.00	19.46	4.57	19.00	29.57	7.10	29.00
No	36	23.84	0.61	1.44	0.67	53.97	8.86	53.50	19.53	4.25	19.00	27.75	6.37	28.00
**Continue training**														
Yes	138	91.39	0.58	1.32	0.67	56.00	10.53	56.00	19.39	4.39	19.00	**29.51**^a^	6.95	29.00
No	13	8.61	0.59	1.38	0.67	51.92	8.81	51.00	20.38	5.52	18.00	25.08	5.88	26.00
**Training program provided**														
Yes	47	31.13	0.57	1.12	0.67	57.32	10.89	58.00	19.34	3.70	19.00	**30.85**^a^	7.59	30.00
No	104	68.87	0.59	1.40	0.67	54.89	10.19	55.00	19.54	4.81	19.00	28.36	6.54	28.00

*M, Mean; SD, Standard Deviation; Mdn, Median*.

The reference category for each dichotomous independent categorical variable is set to “No.”

* * The reference categories are set to Sport Type (Team); Position in Team (Deteriorated a lot)*.

*BEES: Brief Emotional Experience Scale. Lower score indicates lower emotional well-being. Possible score −3 to +3*.

*Coping: Brief COPE Scale. Lower score indicates a poorer ability to cope. Possible score 28–112*.

*Coping avoidant: Brief COPE Domain Scale. Higher score indicates stronger Avoidant coping traits*.

*Coping adaptive: Brief COPE Domain Scale. Higher score indicates stronger Adaptive coping traits*.

a * Significant difference to reference category at p < 0.05*.

b * Sub-category excluded from analysis due to low cell count*.

*Bolded is to emphasize the “a” which denotes: Significant difference to reference category at p < 0.05*.

The occurrence of COVID-19 negatively affected over a third of participant's position security within their sport (position in the sport deteriorated a lot or somewhat) (*N* = 59, 39%) (Table 3). This was similar for individual (*n* = 45, 40% of individual athletes) and team-sport athletes (*n* = 14, 38% of team athletes). Position in sport was not significantly associated with emotional well-being (*p* = 0.206), total coping (*p* = 0.691), avoidant (*p* = 0.247), or adaptive coping (*p* = 0.758) scores, however participants whose position improved somewhat had marginally greater coping, adaptive and avoidant coping scores.

Over half of all participants (*N* = 77, 51%) were formally contacted at least once by all three levels of their sporting organization (National, State, Club) (Table 3). Comparatively, 9% of participants were not formally contacted by anyone from their sporting organization across any level (*N* = 14). Participants not contacted by their national organization had significantly greater mean BEES score (*p* = 0.012) (greater emotional well-being) than participants who were contacted (Table 3). All coping, adaptive and avoidant coping scores were similar between participants contacted by national and participants who were not contacted.

There was no significant difference in emotional well-being, coping, avoidant or adaptive coping scores between participants contacted at a state or club level. However, coping, adaptive and avoidant coping scores were marginally greater for participants contacted by their respective state or club organization.

The majority of participants continued some form of training (*n* = 138, 91%). While total coping scores were greater for participants who continued training, there was no significant difference in total coping scores. Adaptive coping scores were significantly greater for participants able to continue training in comparison to those who were not (0.025).

The majority of participants were not provided by with a training program (*n* = 47, 69%) (Table 3). Overall, whilst not significant, community level athletes had greater coping scores when a training program was provided, compared to when one was not provided. There was no significant difference in emotional well-being, or avoidant coping scores. Participants had a significantly a greater mean adaptive coping score when they had a training program provided (*p* = 0.038) in comparison to those who did not (Table 3).

### Association of Organizational Support on Community Level Athlete's Emotional Well-Being and Coping Ability During COVID-19

Four generalized linear models explored if the level of support provided by a club was associated with a community level athlete's emotional well-being, and ability to cope during COVID-19, while adjusting for other extraneous factors (Table 4).

**Table 4:** Association between the level of support provided by a sport on a community level athletes' emotion and ability to cope during COVID-19 (*N* = 151)^*^.

	**B**	**B 95% CI**	**Sig (*p*-value)**
**MODEL 1. BEES**			
Contacted by club	0.09	−0.43, 0.61	0.738
Contacted by state sporting body	0.05	−0.47, 0.57	0.857
Contacted by national sporting body	−0.63	−1.14, −0.13	**0.014** ^a^
Able to continue training	0.10	−0.64, 0.84	0.787
Provided with a training program	0.06	−0.39, 0.52	0.793
	0.09	−0.43, 0.61	0.738
**MODEL 2. TOTAL COPING**			
Contacted by Club	2.11	−2.07, 6.28	0.322
Contacted by State Sporting Body	1.17	−3.02, 5.37	0.584
Contacted by National Sporting Body	−1.18	−5.30, 2.93	0.573
Able to continue training	3.91	−2.00, 9.83	0.195
Provided with a training program	1.99	−1.65, 5.63	0.283
**MODEL 3. COPING—AVOIDANT**			
Contacted by Club	−0.11	−1.91, 1.69	0.906
Contacted by State Sporting Body	0.17	−1.65, 1.98	0.856
Contacted by National Sporting Body	−0.02	−1.77, 1.74	0.984
Able to continue training	−0.97	−3.53, 1.60	0.459
Provided with a training program	−0.14	−1.72, 1.43	0.858
**MODEL 4. COPING—ADAPTIVE**			
Contacted by club	1.59	−1.12, 4.30	0.249
Contacted by state sporting body	0.41	−2.31, 3.14	0.766
Contacted by national sporting body	−1.05	−3.69, 1.59	0.437
Able to continue training	4.27	0.41, 8.13	**0.030**^a^
Provided with a training program	2.14	−0.23, 4.52	0.077

The reference category for each independent categorical variable is set to “No.”

*No subgroup in the five key independent variables of interest (Level of Support) had a low cell count*.

*Model effect size (R-Squared) is unable to be computed in GenLM using SPSS software*.

*Model 1. Outcome: BEES: Brief Emotional Experience Scale. Lower score indicates lower emotional well-being. Possible score −3 to +3*.

*Model 2. Outcome: Brief COPE Total Scale. Lower score indicates a poorer ability to cope. Possible score 28–112*.

*Model 3. Outcome: Coping Avoidant. Brief COPE Domain Scale. Higher score indicates stronger Avoidant coping traits*.

*Model 4: Outcome: Coping Adaptive. Brief COPE Domain Scale. Higher score indicates stronger Adaptive coping traits*.

a Significant difference at p < 0.05

* *Each model is adjusted for state or territory, sex, age, change in employment status due to COVID-19, and medical condition (respiratory, immune compromised, other), team or Individual sport and position security within the sport*.

*Bolded is to emphasize the “a” which denotes: Significant difference to reference category at p < 0.05*.

Model 1 indicated contact from national sporting body was significantly associated with poorer emotional well-being (B = −0.63, 95% CI: −1.14, −0.13; *p* = 0.014) when adjusting for demographic and other sporting factors. No other level of support was associated with emotional well-being.

In Model 2, when adjusting for demographic and other sporting factors, the level of support provided was not associated with a community level athlete's ability to cope during COVID-19. Similarly, in Model 3, the level of support provided to an athlete was not associated with avoidant coping, when adjusting for demographic and other sporting factors. Model 4 indicated the ability to continue training was significantly associated with greater adaptive coping scores (B = 4.27, 95% CI: 0.41, 8.13; *p* = 0.033) when adjusting for demographic and other sporting factors. However, the wide confidence intervals need to be acknowledged, meaning a large plausible range in adaptive coping scores. Other variables reflecting level of support were not significantly associated with adaptive coping score outcomes.

## Discussion

COVID-19 has impacted the way of life of individuals worldwide, leading to a deterioration in emotional well-being, with several studies, including those in athletic populations, reporting an increase in stress, anxiety and depressive symptomology (Cullen et al., 2020; Holmes et al., 2020; Pierce et al., 2020; Reardon et al., 2020). Few studies have investigated the implications of COVID-19 on community level athletes (Asif et al., 2020; Mann et al., 2020) and the ability of athletes to cope during the pandemic (Makarowski et al., 2020). Given earlier findings (Adami et al., 2020; di Fronso et al., 2020; Mann et al., 2020; Pillay et al., 2020), the protective role of sport engagement on mental health (Vella et al., 2019), and the imposed restrictions on sport engagement, researchers anticipated that Australian community level athletes would experience a deterioration in emotional well-being, which would be related to a diminished capacity to employ protective coping strategies. In contrast to our expectations, we found no evidence of a decrease in emotional well-being or poorer use of coping strategies between Australian community athletes during the COVID-19 pandemic.

While the coping capacity of community athletes in Australia was found to be similar in community athletes irrespective of demographics or level of sporting support received, this finding could be related to the fewer positive COVID-19 cases and associated deaths compared with many other countries worldwide (Communicable Diseases Intelligence: COVID-19 National Incident Room Surveillance Team, 2020). Additionally, Australia had shorter lockdown periods, therefore limiting in-person physical distancing and social isolation, which are known to adversely impact on mental health and coping mechanisms (Smith and Victor, 2019; Usher et al., 2020). However, with the cross-sectional study design, further research would be required to ascertain if these coping scores are unique to the Australian COVID-19 setting, lower infection and mortality rates, or impacted directly by the pandemic Other Australian-centric factors were likely to have contributed to the outcomes of this study. For instance, while coping was seemingly greater for participants who lost employment, it is noted that the Australian Federal Government swiftly provided more robust and pandemic-specific national support to anyone that lost employment (via *Job Seeker*) or was temporarily displaced (for businesses via *Job Keeper*) as a result of COVID-19 to ensure temporary financial stability. Additionally, Australian athletes were able to continue training throughout the nation under varied circumstances (e.g., decreased numbers, non-contact, web-based) and maintained a sense of community throughout the lockdown process as a result.

Adaptive Coping scores were higher for females compared to males, which may be aligned with women being more likely to engage in help-seeking behaviors (Liddon et al., 2018; Volk et al., 2020). Further analysis of coping scores across states and between individual and team sports, revealed that participation in any community level sporting type or location yielded positive coping results. These findings were unexpected given early reports of greater anxiety and depressive symptomology in athletes who had trained in a club facility (Makarowski et al., 2020; McGee and Sanders, 2020). This was presumed to be due to, at least in part, an inability to cope with the indirect impacts of COVID-19 on employment, training and participating in competitive matches.

There is a well-documented relationship between utilizing better coping strategies/mechanisms and experiencing improved emotional well-being (Berto, 2014; Tremolada et al., 2016; Marroquín et al., 2017). Both adaptive (e.g., seeking social support, acceptance) and maladaptive (e.g., rumination, avoidance) coping strategies are part of the human reaction to adverse experiences and are affiliated with greater emotional well-being (Marroquín et al., 2017). Despite higher coping scores being reported by female athletes in the present study, they contrastingly reported lower emotional well-being scores than their male counterparts. This phenomenon can be attributed to female athletes tending to ruminate more (di Fronso et al., 2020), report less access to training equipment during the lockdown (Bowes et al., 2020), as well as being more likely to perceive the pandemic as a serious health problem thereby potentially being more compliant with physical distancing and social isolation measures (Galasso et al., 2020; Liu et al., 2020). Women continue to be disproportionately affected by the COVID-19 pandemic—they are more susceptible to increased rates of intimate partner violence (Connor et al., 2020; Mazza et al., 2020), have seen an increase in burden of care (e.g., household responsibilities Fodor et al., 2020; Kibbe, 2020), and have a higher presence on the frontlines (e.g., nurses Bahri, 2020; McLaren et al., 2020).

Although not significant, emotional well-being was greater in participants whose employment was not affected, as job instability is associated with worse mental health outcomes (Moen et al., 2020; Wilson et al., 2020). Similarly, not significant but of note, respondents that identified as being part of a team sport had marginally greater emotional well-being scores. This is likely attributed to the emphasis on staying connected with teammates (Jukic et al., 2020) and the call-to-action to make a concerted effort to reintroduce group activities for emotional well-being (Denay et al., 2020; Hughes et al., 2020).

The longer-term psychological impact of COVID-19 is widely speculated (Butler and Barrientos, 2020; Sher, 2020; Talevi et al., 2020) as Australia and countries around the world continue to endure subsequent COVID-19 outbreaks and affiliated implications (i.e., extended regulations to maintain physical distancing and social isolation). Ensuring training opportunities are available and modifiable for community level athletes will preserve coping capacity and further strengthen their emotional well-being (Strohle, 2019; Teychenne et al., 2020). Therefore, additional research will assist with the potential lasting effects of the global pandemic (Kathirvel, 2020; Moreno et al., 2020) as the world of returning to community sport hangs in the balance (Ranasinghe et al., 2020).

### Limitations

A number of limitations need to be considered when interpreting the study findings. First, the study only included individuals within Australia, where the transmission of COVID-19 has been comparably lower than the vast majority of other countries and individuals were still allowed to exercise, provided they adhered to physical distancing. Additionally, prospective pre-COVID coping and emotional well-being scores were not available, therefore conclusions cannot be drawn about changes in these outcomes, and a causal link to COVID-19 cannot be established with the current study design.

Furthermore, the findings may not be generalisable to all community athletes due to the low sample size, however. these findings offer an initial insight to coping and emotional well-being in athletes within Australia. Australian states and territories were disproportionately impacted by COVID-19, with varying lock down requirements and durations. The final models attempted to control for this by adjusting based on state with low cell counts and wide beta confidence intervals, however residuals were normal. Next, the results may have been influenced by sampling (e.g., responder) bias, with more mentally fit participants more likely to complete the survey. In addition, the large number of Western Australian and female participants could also skew results, while increased alcohol use throughout Australia (Neill et al., 2020) may be confounding factors. Finally, this study did not exclude or control for people with pre-existing mental health conditions. Given the nature of the survey and speed of COVID-19 transmission this was not feasible.

## Conclusion

It was anticipated that the implications of COVID-19 (e.g., uncertainty on return to sport conditions, athlete safety) would have significant detrimental effects on community-level athlete well-being and their ability to cope. Here we report no differences in coping or emotional well-being between demographic factors, or based on the level of support received in an Australian cohort of community athletes. While these findings are undoubtedly of interest, there is a fundamental need for researchers to investigate the longer-term ramifications of COVID-19 on community level athletes in Australia and worldwide.

## Data Availability Statement

The raw data supporting the conclusions of this article will be made available by the authors, without undue reservation.

## Ethics Statement

The studies involving human participants were reviewed and approved by Edith Cowan University Human Research Ethics Committee HREC: 2020-01315. The patients/participants provided their written informed consent to participate in this study.

## Author Contributions

CF-H: investigation and writing—original draft. SH: formal analysis and writing—original draft. SR: conceptualization, methodology, and validation. SV and PB: conceptualization, methodology, investigation, and validation. MT: conceptualization, methodology, investigation, data curation, and writing—original draft. TC: conceptualization, methodology, investigation, data curation, and writing—original draft. All authors contributed to the article and approved the submitted version.

## Conflict of Interest

The authors declare that the research was conducted in the absence of any commercial or financial relationships that could be construed as a potential conflict of interest.

## References

[B1] Adami P. E. Cianca J. McCloskey B. Derman W. Steinacker J. M. O'Connor F.. (2020). Infectious Diseases Outbreak Management Tool for endurance mass participation sporting events: an international effort to counteract the COVID-19 spread in the endurance sport setting. Br. J. Sports Med. 55, 181–182. 10.1136/bjsports-2020-103091 32819919

[B2] Anshel M. H. Kaissidis A. N. (1997). Coping style and situational appraisals as predictors of coping strategies following stressful events in sport as a function of gender and skill level. Br. J. Psychol. 88, 263–276. 10.1111/j.2044-8295.1997.tb02634.x 9183840

[B3] Anshel M. H. Sutarso T. Jubenville C. (2009). Racial and gender differences on sources of acute stress and coping style among competitive athletes. J. Soc. Psychol. 149, 159–178. 10.3200/SOCP.149.2.159-178 19425355

[B4] Asif I. M. Chang C. J. Diamond A. B. Raukar N. Zaremski J. L. (2020). Returning athletes back to high school sports in the COVID-19 era: preparing for the fall. Sports Health. 12, 518–520. 10.1177/1941738120953851 32816648 PMC7785895

[B5] Bahri A. (2020). Women at the Frontline of COVID-19: Can Gender Mainstreaming in Free Trade Agreements Help? J. Int. Econ. Law. 23, 563–582. 10.1093/jiel/jgaa023

[B6] Berto R. (2014). The role of nature in coping with psycho-physiological stress: a literature review on restorativeness. Behav. Sci. (Basel). 4, 394–409. 10.3390/bs4040394 25431444 PMC4287696

[B7] Bowes A. Lomax L. Piasecki J. (2020). The impact of the COVID-19 lockdown on elite sportswomen. Manag. Sport Leisure 1–17. 10.1080/23750472.2020.1825988 Available online at: https://www-tandfonline-com.ezproxy.ecu.edu.au/doi/pdf/10.1080/23750472.2020.1825988?needAccess=true

[B8] Brown S. P. Westbrook R. A. Challagalla G. (2005). Good cope, bad cope: adaptive and maladaptive coping strategies following a critical negative work event. J. Appl. Psychol. 90, 792–798. 10.1037/0021-9010.90.4.792 16060796

[B9] Butler M. J. Barrientos R. M. (2020). The impact of nutrition on COVID-19 susceptibility and long-term consequences. Brain Behav. Immun. 87, 53–54. 10.1016/j.bbi.2020.04.040 32311498 PMC7165103

[B10] Carver C. S. (1997). You want to measure coping but your protocol'too long: consider the brief cope. Int. J. Behav. Med. 4:92. 10.1207/s15327558ijbm0401_6 16250744

[B11] Cheng C. Ebrahimi O. V. Lau Y. C. (2020). Maladaptive coping with the infodemic and sleep disturbance in the COVID-19 pandemic. J. Sleep Res. e13235. 10.1111/jsr.13235 Available online at: https://onlinelibrary.wiley.com/doi/epdf/10.1111/jsr.13235 PMC7744904 33247519

[B12] Communicable Diseases Intelligence: COVID-19 National Incident Room Surveillance Team (2020). COVID-19, Australia: epidemiology report 20 (fortnightly reporting period ending 5 July 2020). Canberra.

[B13] Connor J. Madhavan S. Mokashi M. Amanuel H. Johnson N. R. Pace L. E.. (2020). Health risks and outcomes that disproportionately affect women during the Covid-19 pandemic: a review. Soc. Sci. Med. 266:113364. 10.1016/j.socscimed.2020.113364 32950924 PMC7487147

[B14] Cullen W. Gulati G. Kelly B. D. (2020). Mental health in the Covid-19 pandemic. QJM. 113, 311–312. 10.1093/qjmed/hcaa110 32227218 PMC7184387

[B15] Denay K. L. Breslow R. G. Turner M. N. Nieman D. C. Roberts W. O. Best T. M. (2020). ACSM call to action statement: COVID-19 considerations for sports and physical activity. Curr. Sports Med. Rep. 19, 326–328. 10.1249/JSR.0000000000000739 32769667

[B16] di Fronso S. Costa S. Montesano C. Di Gruttola F. Ciofi E. G. Morgilli L.. (2020). The effects of COVID-19 pandemic on perceived stress and psychobiosocial states in Italian athletes. Int. J. Sport Exerc. Psychol. 1–13. 10.1080/1612197X.2020.1802612 Available online at: https://www.tandfonline.com/doi/pdf/10.1080/1612197X.2020.1802612?needAccess=true

[B17] Fitzpatrick K. M. Harris C. Drawve G. (2020). Fear of COVID-19 and the mental health consequences in America. Psychol. Trauma. 12, S17–S21. 10.1037/tra0000924 32496100

[B18] Fodor É. Gregor A. Koltai J. Kováts E. (2020). The impact of COVID-19 on the gender division of childcare work in Hungary. Europ. Soc. 23, S95–S110. 10.1080/14616696.2020.1817522

[B19] Galasso V. Pons V. Profeta P. Becher M. Brouard S. Foucault M. (2020). Gender differences in COVID-19 attitudes and behavior: Panel evidence from eight countries. Proc. Natl. Acad. Sci. U.S.A. 117, 27285–27291. 10.1073/pnas.2012520117 33060298 PMC7959517

[B20] Galea S. Merchant R. M. Lurie N. (2020). The mental health consequences of COVID-19 and physical distancing: The need for prevention and early intervention. JAMA Intern. Med. 180, 817–818. 10.1001/jamainternmed.2020.1562 32275292

[B21] Holmes E. A. O'Connor R. C. Perry V. H. Tracey I. Wessely S. Arseneault L.. (2020). Multidisciplinary research priorities for the COVID-19 pandemic: a call for action for mental health science. Lancet Psychiatry. 7, 547–560. 10.1016/S2215-0366(20)30168-1 32304649 PMC7159850

[B22] Hughes D. Saw R. Perera N. K. P. Mooney M. Wallett A. Cooke J.. (2020). The Australian Institute of Sport framework for rebooting sport in a COVID-19 environment. J. Sci. Med. Sport. 23, 639–663. 10.1016/j.jsams.2020.05.004 32451268 PMC7200343

[B23] Jukic I. Calleja-González J. Cos F. Cuzzolin F. Olmo J. Terrados N.. (2020). Strategies and Solutions for Team Sports Athletes in Isolation due to COVID-19. Sports. 8:56. 10.3390/sports8040056 32344657 PMC7240607

[B24] Kathirvel N. (2020). Post COVID-19 pandemic mental health challenges. Asian J. Psychiatr. 53:102530. 10.1016/j.ajp.2020.102430 PMC7507979 33264840

[B25] Kibbe M. R. (2020). Consequences of the COVID-19 pandemic on manuscript submissions by women. JAMA Surg. 155, 803–804. 10.1001/jamasurg.2020.3917 32749449

[B26] Kumari S. Mahla R. S. (2020). Stay-at-home isolation modulates sleep pattern associating with depression and anxiety mood disorders. J. Clin. Sleep Med. 17, 343–344. 10.5664/jcsm.8908 33094726 PMC7853208

[B27] Lazzerini M. Barbi E. Apicella A. Marchetti F. Cardinale F. Trobia G. (2020). Delayed access or provision of care in Italy resulting from fear of COVID-19. Lancet Child Adol. Health. 4, e10–e11. 10.1016/S2352-4642(20)30108-5 32278365 PMC7146704

[B28] Liddon L. Kingerlee R. Barry J. A. (2018). Gender differences in preferences for psychological treatment, coping strategies, and triggers to help-seeking. Br. J. Clin. Psychol. 57, 42–58. 10.1111/bjc.12147 28691375

[B29] Liu N. Zhang F. Wei C. Jia Y. Shang Z. Sun L.. (2020). Prevalence and predictors of PTSS during COVID-19 outbreak in China hardest-hit areas: Gender differences matter. Psychiatry Res. 287:112921. 10.1016/j.psychres.2020.112921 32240896 PMC7102622

[B30] Ludvigsen J. A. L. Hayton J. W. (2020). Toward COVID-19 secure events: Considerations for organizing the safe resumption of major sporting events. Manag. Sport Leisure 35, 557–568. 10.1080/23750472.2020.1782252

[B31] Mahmoud J. S. R. Staten R. T. Hall L. A. Lennie T. A. (2012). The relationship among young adult college students' depression, anxiety, stress, demographics, life satisfaction, and coping styles. Issues Ment. Health Nurs. 33, 149–156. 10.3109/01612840.2011.632708 22364426

[B32] Makarowski R. Piotrowski A. Predoiu R. Görner K. Predoiu A. Mitrache G.. (2020). Stress and coping during the COVID-19 pandemic among martial arts athletes–a cross-cultural study. Arch. Budo. 16, 161–171. Available online at: https://vb.lsu.lt/object/elaba:72060534/

[B33] Mann R. H. Clift B. C. Boykoff J. Bekker S. (2020). Athletes as community; athletes in community: covid-19, sporting mega-events and athlete health protection. Br. J. Sports Med. 54, 1071–1072. 10.1136/bjsports-2020-102433 32303522 PMC7497562

[B34] Marroquín B. Tennen H. Stanton A. L. (2017). “Coping, emotion regulation, and well-being: Intrapersonal and interpersonal processes,” in The Happy Mind: Cognitive Contributions to Well-Being, eds Robinson MD Eid E (Springer International Publishing AG), 253–274. 10.1007/978-3-319-58763-9_14

[B35] May C. (2020). Clearinghouse: Sport Participation In Australia. [online] Clearinghouseforsport.gov.au. Available online at: https://www.clearinghouseforsport.gov.au/kb/sport-participation-in-australia.

[B36] Mazza M. Marano G. Lai C. Janiri L. Sani G. (2020). Danger in danger: Interpersonal violence during COVID-19 quarantine. Psychiatry Res. 289:113046. 10.1016/j.psychres.2020.113046 32387794 PMC7190494

[B37] McGee U. Sanders E. (2020). Letter to the editor regarding the effect of isolation on athletes' mental health during the COVID-19 pandemic. Physic. Sportsmedi. 1, 1–1. 10.1080/00913847.2020.1845582 33148087

[B38] McGuine T. A. Biese K. M. Petrovska L. Hetzel S. J. Reardon C. Kliethermes S.. (2020a). Mental health, physical activity, and quality of life of US adolescent athletes during COVID-19–related school closures and sport cancellations: a study of 13 000 athletes. J. Athl. Train. 56, 11–19. 10.4085/1062-6050-0478.20 33290516 PMC7863599

[B39] McGuine T. A. Biese K. M. Petrovska L. Hetzel S. J. Reardon C. Kliethermes S.. (2020b). The health of US adolescent athletes during Covid-19 related school closures and sport cancellations. J. Athlet. Train. 56, 11–19. 10.4085/478-20 33290516 PMC7863599

[B40] McLaren H. J. Wong K. R. Nguyen K. N. Mahamadachchi K. N. D. (2020). Covid-19 and Women's Triple Burden: Vignettes from Sri Lanka, Malaysia, Vietnam and Australia. Soc. Sci. 9:87. 10.3390/socsci9050087

[B41] Moen P. Pedtke J. H. Flood S. (2020). Disparate disruptions: Intersectional COVID-19 employment effects by age, gender, education, and race/ethnicity. Work Aging Retir. 6, 207–228. 10.1093/workar/waaa013 33214905 PMC7543645

[B42] Montemurro N. (2020). The emotional impact of COVID-19: From medical staff to common people. Brain Behav Immun. 87, 23–24. 10.1016/j.bbi.2020.03.032 32240766 PMC7138159

[B43] Moreno C. Wykes T. Galderisi S. Nordentoft M. Crossley N. Jones N.. (2020). How mental health care should change as a consequence of the COVID-19 pandemic. Lancet Psychiatry 7 813–824. 10.1016/S2215-0366(20)30307-2 32682460 PMC7365642

[B44] Murray M. T. Riggs M. A. Engelthaler D. M. Johnson C. Watkins S. Longenberger A.. (2020). Mitigating a COVID-19 outbreak among major league baseball players—United States, 2020. Morbid. Mortal. Weekly Report. 69, 1542–1546. 10.15585/mmwr.mm6942a4 33090983 PMC7583504

[B45] Neill E. Meyer D. Toh W. L. van Rheenen T. E. Phillipou A. Tan E. J.. (2020). Alcohol use in Australia during the early days of the COVID-19 pandemic: Initial results from the COLLATE project. Psychiatry Clin. Neurosci. 74, 542–549. 10.1111/pcn.13099 32602150 PMC7436134

[B46] Ozamiz-Etxebarria N. Idoiaga Mondragon N. Dosil Santamaría M. Picaza Gorrotxategi M. (2020). Psychological symptoms during the two stages of lockdown in response to the COVID-19 outbreak: an investigation in a sample of citizens in Northern Spain. Front. Psychol. 11, 1–9. 10.3389/fpsyg.2020.01491 32625157 PMC7314923

[B47] Pfefferbaum B. North C. S. (2020). Mental health and the Covid-19 pandemic. N. Engl. J. Med. 383, 510–512. 10.1056/NEJMp2008017 32283003

[B48] Pierce M. Hope H. Ford T. Hatch S. Hotopf M. John A.. (2020). Mental health before and during the COVID-19 pandemic: a longitudinal probability sample survey of the UK population. Lancet Psychiatry. 7, 883–892. 10.1016/S2215-0366(20)30308-4 32707037 PMC7373389

[B49] Pillay L. van Rensburg D. C. C. J. van Rensburg A. J. Ramagole D. A. Holtzhausen L. Dijkstra H. P.. (2020). Nowhere to hide: the significant impact of coronavirus disease 2019 (COVID-19) measures on elite and semi-elite South African athletes. J. Sci. Med. Sport. 23, 670–679. 10.1016/j.jsams.2020.05.016 32448749 PMC7235602

[B50] Ranasinghe C. Jayawardena R. Palihawadana V. (2020). Kick start training during the COVID-19 pandemic-Challenges of the sporting community. Sri Lankan J. Sports Exerc. Med. 2, 1–9. 10.4038/sljsem.v2i1.17

[B51] Reardon C. L. Bindra A. Blauwet C. Budgett R. Campriani N. Currie A.. (2020). Mental health management of elite athletes during COVID-19: a narrative review and recommendations. Br. J. Sports Med. 10.1136/bjsports-2020-102884 Available online at: https://bjsm-bmj-com.ezproxy.ecu.edu.au/content/bjsports/early/2020/09/23/bjsports-2020-102884.full.pdf 32967853

[B52] Rettie H. Daniels J. (2020). Coping and tolerance of uncertainty: Predictors and mediators of mental health during the COVID-19 pandemic. Am. Psychol. 10.1037/amp0000710 Available online at: http://web.b.ebscohost.com.ezproxy.ecu.edu.au/ehost/pdfviewer/pdfviewer?vid=1&sid=2939583f-1200-45c6-894a-5b43533ccb1b%40sessionmgr101 32744841

[B53] Rogers S. L. Barblett L. Robinson K. (2016). Investigating the impact of NAPLAN on student, parent and teacher emotional distress in independent schools. Austr. Educ. Res. 43, 327–343. 10.1007/s13384-016-0203-x

[B54] Rogers S. L. Cruickshank T. (2020). Change in mental and physical health, and social relationships, during highly restrictive lockdown in the COVID-19 pandemic: evidence from Australia. Psyarxiv [preprint]. 10.31234/osf.io/zutav PMC8310621 34327055

[B55] Rossi R. Socci V. Talevi D. Mensi S. Niolu C. Pacitti F.. (2020). COVID-19 pandemic and lockdown measures impact on mental health among the general population in Italy. Front Psychiatry 11:790. 10.3389/fpsyt.2020.00790 32848952 PMC7426501

[B56] Shanahan L. Steinhoff A. Bechtiger L. Murray A. L. Nivette A. Hepp U.. (2020). Emotional distress in young adults during the COVID-19 pandemic: evidence of risk and resilience from a longitudinal cohort study. Psychol. Med. 1–10. 10.1017/S003329172000241X Available online at: https://www.cambridge.org/core/journals/psychological-medicine/article/emotional-distress-in-young-adults-during-the-covid19-pandemic-evidence-of-risk-and-resilience-from-a-longitudinal-cohort-study/BD42C8C4EDFEEC6255554B195EA4ADED PMC7338432 32571438

[B57] Sher L. (2020). The impact of the COVID-19 pandemic on suicide rates. QJM. 113, 707–712. 10.1093/qjmed/hcaa202 32539153 PMC7313777

[B58] Skead N. K. Rogers S. L. (2016). Running to well-being: A comparative study on the impact of exercise on the physical and mental health of law and psychology students. Int. J. Law Psychiatry 49, 66–74. 10.1016/j.ijlp.2016.05.012 27241463

[B59] Skead N. K. Rogers S. L. Doraisamy J. (2018). Looking beyond the mirror: Psychological distress; disordered eating, weight and shape concerns; and maladaptive eating habits in lawyers and law students. Int. J. Law Psychiatry. 61, 90–102. 10.1016/j.ijlp.2018.06.002 30219481

[B60] Skead N. K. Rogers S. L. Johnson W. R. (2020). The role of place, people and perception in law student well-being. Int. J. Law Psychiatry 73:101631. 10.1016/j.ijlp.2020.101631 33027699

[B61] Smith K. J. Victor C. (2019). Typologies of loneliness, living alone and social isolation, and their associations with physical and mental health. Ageing Soc. 39, 1709–1730. 10.1017/S0144686X18000132

[B62] Staples L. Nielssen O. Kayrouz R. Cross S. Karin E. Ryan K.. (2020). Rapid report 2: Symptoms of anxiety and depression during the first 12 weeks of the Coronavirus (COVID-19) pandemic in Australia. Internet Intervent. 22:100351. 10.1016/j.invent.2020.100351 33110762 PMC7580521

[B63] Strohle A. (2019). Sports psychiatry: mental health and mental disorders in athletes and exercise treatment of mental disorders. Eur. Arch. Psychiatry Clin. Neurosci. 269, 485–498. 10.1007/s00406-018-0891-5 29564546

[B64] Talevi D. Socci V. Carai M. Carnaghi G. Faleri S. Trebbi E.. (2020). Mental health outcomes of the CoViD-19 pandemic. Riv. Psichiatr. 55, 137–144. 10.1708/3382.33569 32489190

[B65] Teychenne M. White R. L. Richards J. Schuch F. B. Rosenbaum S. Bennie J. A. (2020). Do we need physical activity guidelines for mental health: What does the evidence tell us?. Ment. Health Phys. Act. 18:100315. 10.1016/j.mhpa.2019.100315

[B66] Tremolada M. Bonichini S. Taverna L. (2016). Coping strategies and perceived support in adolescents and young adults: predictive model of self-reported cognitive and mood problems. Psychology 9, 1858–1871. 10.4236/psych.2016.714171

[B67] Usher K. Bhullar N. Jackson D. (2020). Life in the pandemic: social isolation and mental health. J. Clin. Nurs. 29, 2756–2757. 10.1111/jocn.15290 32250493

[B68] Van Rheenen T. E. Meyer D. Neill E. Phillipou A. Tan E. J. Toh W. L.. (2020). Mental health status of individuals with a mood-disorder during the COVID-19 pandemic in Australia: Initial results from the COLLATE project. J. Affect. Disord. 275, 69–77. 10.1016/j.jad.2020.06.037 32658826 PMC7331562

[B69] Vella S. A. Swann C. Boydell K. M. Eckermann S. Fogarty A. Hurley D.. (2019). Sports-based mental health promotion in Australia: formative evaluation. Psychol. Sport Exerc. 45:101560. 10.1016/j.psychsport.2019.101560

[B70] Volk A. A. Brazil K. J. Franklin-Luther P. Dane A. V. Vaillancourt T. (2020). The influence of demographics and personality on COVID-19 coping in young adults. Pers. Individ. Dif. 168:110398. 10.1016/j.paid.2020.110398 32952250 PMC7492069

[B71] Wilson J. M. Lee J. Fitzgerald H. N. Oosterhoff B. Sevi B. Shook N. J. (2020). Job insecurity and financial concern during the COVID-19 pandemic are associated with worse mental health. J. Occup. Environ. Med. 62, 686–691. 10.1097/JOM.0000000000001962 32890205

