# Supplementary material for: A Survey to Evaluate the Association of COVID-19 Restrictions on Perceived Mood and Coping in Australian Community Level Athletes
Source: Front Sports Act Living. 2021 Mar 22;3:624267. doi: 10.3389/fspor.2021.624267 (PMC8021018; doi:10.3389/fspor.2021.624267)
Supplement: Supplementary file 1 [file Data_Sheet_1.PDF]

COVID-19 Athlete Survey

Start of Block: COVID-19 well-being survey

COVID-19 athlete survey -

Dear participant, PLEASE NOTE: THIS RESEARCH IS ONLY APPLICABLE TO PEOPLE WHO ARE 18 YEARS OR OVER, AND WHO ARE RESIDING IN AUSTRALIA. The COVID-19 pandemic is currently having impacts around the entire world. This survey is designed to gather information on how it is impacting Australian athletes. The survey will ask questions regarding: - Demographic information about your age, gender, and employment - Impact of COVID-19 - The effect of COVID-19 on your: - Emotional wellbeing - Sporting activities - Sporting entities - Sleep quality Answering this survey in its entirety should take a total of 15-20 minutes to complete. All data collected will be stored securely and confidentiality will be maintained, as all responses made are anonymous. There is the potential that collected data will be used for publication purposes, that will include posting the raw data file onto an online research repository. Since this survey is anonymous, upon completion your responses will not be able to be withdrawn. To obtain a copy of the general results of the research please contact Travis Cruickshank. There is no monetary incentive for completing this survey. The research project has been approved by the ECU Human Research Ethics Committee. If you have any concerns or complaints regarding the research project and would like to speak to an independent person, please contact the Research Ethics Officer on 6304 2170 or via email research.ethics@ecu.edu.au Sincerely yours, Dr. Travis Cruickshank Dr. Travis Cruickshank School of Medical and Health Sciences Edith Cowan University, Joondalup Campus, WA Email: t.cruickshank@ecu.edu.au

---

Consent

I have read the above information about the study and I understand that: - If I have any additional questions I can contact the lead researcher.- In this survey no identifying information is collected so for all intents and purposes responses are anonymous.- I am free to withdraw from further participation at any time, without explanation or penalty.- If I complete the survey and submit, then I am unable to withdraw my responses since the responses are anonymous. -

I freely agree to participate in the project.   Please indicate whether or not you consent to participate in this research:

◯ Yes, I consent for my responses to be used for research purposes (1)

◯ No, I do not consent to participate (*If you choose this option please close down your browser and do not proceed) (2)

*Skip To: End of Survey If Consent   I have read the above information about the study and I understand that: - If I have... = No, I do not consent to participate (*If you choose this option please close down your browser and do not proceed)*

Mental health resources   *This survey includes questions that ask about the impact of COVID-19 pandemic on your livelihood and physical and emotional well-being. If completing this survey makes you feel like you would like to talk to someone about any negative feelings that you might be experiencing, please be aware there are options available.*   In Australia, see links below to web pages which all list potential services (*If looking at any of these links please right-click on them and 'open in a new window', or in a new tab, so that the current page stays open).   Government mental health web page: https://www.mentalhealth.gov/get-help LifeLine: https://www.lifeline.org.au/get-help/get-help-home Find a psychologist via Australian Psychological Society: https://www.psychology.org.au/Find-a-Psychologist Find a registered counsellor via the Australian Counselling Association: https://www.theaca.net.au/find-registered-counsellor.phpCOVID-19 support resource: https://headtohealth.gov.au/covid-19-support/covid-19
Please note these same links are also provided at the end of this survey.

To continue with the survey please click 'next' button.

End of Block: COVID-19 well-being survey

Start of Block: Demographics

Q1 My state/territory of residence:

▼ ACT (1) ... WA (8)

Q2 My gender:

- ○ Male (1)
- ○ Female (2)
- ○ Other (3)
- ○ I would prefer not to say (4)

Q3 My age:

▼ 18-21 years (1) ... 71 years or older (9)

Q4 My relationship status:

- ○ I am single (1)
- ○ I am presently in a committed relationship (2)
- ○ Other (4)

Q5 My main sport:

- ○ Archery (24)
- ○ Australian Rules Football (1)
- ○ Badminton (27)
- ○ Basketball (7)
- ○ Cricket (4)
- ○ Cycling (9)
- ○ Dancing (11)
- ○ Diving (25)
- ○ Fencing (17)
- ○ Golf (16)
- ○ Gymnastics (19)
- ○ Martial arts (12)
- ○ Netball (6)
- ○ Rugby League (2)
- ○ Rugby Union (3)
- ○ Running (15)
- ○ Tennis (5)
- ○ Shooting (23)
- ○ Soccer (8)
- ○ Squash (26)
- ○ Swimming (10)

○ Track and field (14)

○ Other (please specify) (28) ____

Q6 My current sporting status:

○ Professional (full-time paid athlete) (1)

○ Semi-professional (paid athlete with additional employment) (2)

○ Community level (not a paid athlete) (3)

Skip To: Q7 If My current sporting status: = Professional (full-time paid athlete)

Q6a My employment status prior to COVID-19 (outside of athlete employment):

*Note: This question also refers to self-employment. So for example if you work for yourself on full-time basis you would select 'full-time'.

▼ Full-time (1) ... Unemployed (5)

Display This Question:

*If My employment status prior to COVID-19 (outside of athlete employment): *Note: This question also... = Full-time*

*Or My employment status prior to COVID-19 (outside of athlete employment): *Note: This question also... = Part-time*

*Or My employment status prior to COVID-19 (outside of athlete employment): *Note: This question also... = Casual*

Q6b The industry I classify my work as belonging to is:

▼ Chief Executives, General Managers and Legislators (1) ... Other Labourers (41)

Q7 I have lost employment due to the COVID-19 situation:

○ Yes (1)

○ No (3)

Display This Question:

If I have lost employment due to the COVID-19 situation: = No

Q7a To what extent has your employment decreased since the COVID-19 situation?

▼ 90% (2) ... 0% (11)

Q8 I **have a medical condition that impacts the following physiological system/s** (select all that apply):

- ▢ Circulatory system (1)
- ▢ Digestive system (2)
- ▢ Endocrine system (3)
- ▢ Exocrine system (i.e., skin, hair, nails, sweat) (4)
- ▢ Immune/lymphatic system (5)
- ▢ Muscular system (6)
- ▢ Nervous system (7)
- ▢ Renal/urinary system (8)
- ▢ Reproductive system (9)
- ▢ Respiratory system (10)
- ▢ Skeletal system (11)
- ▢ Other medical condition not listed here (12)
- ▢ I do not have a medical condition (13)

Q9 If your sport was not impacted by COVID-19 would you be able to compete?

- ○ Yes (1)
- ○ No (3)

Display This Question:

If If your sport was not impacted by COVID-19 would you be able to compete? = No

Q9a Why were you not able to compete?

◯ Injury (2)

◯ Suspension (3)

◯ Other (4) ____

End of Block: Demographics

Start of Block: Impact

Q1 On average, since the emergence of the COVID-19 situation I have been contacted by:

	Never (1)	Once a month (2)	Once every 2 weeks (4)	Once a week (5)	Every few days (7)	Once a day (6)
My national sporting body (e.g., Tennis Australia) (1)	◯	◯	◯	◯	◯	◯
My state sporting body (e.g., Tennis Queensland) (2)	◯	◯	◯	◯	◯	◯
My local sporting club (e.g. Toowoomba Tennis Club) (3)	◯	◯	◯	◯	◯	◯

Page Break

Q2 COVID-19 has impacted my:

	Deteriorated a lot (1)	Deteriorated somewhat (2)	No change (3)	Improved somewhat (4)	Improved a lot (5)
Mental health (1)	o	o	o	o	o
Physical health (2)	o	o	o	o	o
Financial situation (3)	o	o	o	o	o
Sporting situation (position in team) (6)	o	o	o	o	o
Relationships with family (4)	o	o	o	o	o
Relationships with friends (5)	o	o	o	o	o
Relationships with members in my sport (8)	o	o	o	o	o

Page Break

Q3 Overall, how would you rate the impact of the COVID-19 situation on you personally?

▼ Has not impacted me at all (1) ... Prefer not to say (5)

Q4 How long do you think the COVID-19 situation will last?

▼ A few more weeks (1) ... I am very uncertain about how long it will last (8)

Q5 Have you been notified to cease all sporting activities?

◯ Yes (1)

◯ No (2)

Display This Question:

If Have you been notified to cease all sporting activities? = Yes

Q5a When did you receive the notification to cease sporting activities?
Date: DD/MM

____

Q6 Are you currently performing any type of physical training?

◯ Yes (1)

◯ No (2)

Display This Question:

If Are you currently performing any type of physical training? = Yes

Q6a Is the physical training you are performing sports specific?

◯ Yes (1)

◯ No (2)

Display This Question:

If Are you currently performing any type of physical training? = Yes

Q6b To what extent has your training decreased since the COVID-19 situation?

▼ 90% (5) ... 0% (14)

Display This Question:

If Are you currently performing any type of physical training? = Yes

Q6c Where are you performing your physical training?

◯ Home (1)

◯ Local park (3)

◯ Sporting club (4)

◯ Other (5) ____

Display This Question:

If Are you currently performing any type of physical training? = Yes

Q6d What equipment can you access for your physical training?

- ☐ Bodyweight (1)
- ☐ Free weights (2)
- ☐ Bands/tubes (3)
- ☐ Resistance Machines (e.g. leg press) (4)
- ☐ Cardio machines (e.g. treadmill) (6)
- ☐ Other (5) ____

Q7 Have you been provided with a physical training program from your sporting club / state sporting body / national sporting body?

- ○ Yes (1)
- ○ No (2)

Q8 Is your sporting club / state sporting body / national sporting body monitoring your training?

- ○ Yes (1)
- ○ No (2)

Display This Question:

If Is your sporting club / state sporting body / national sporting body monitoring your training? = Yes

Q8a How frequently is your sporting club / state sporting body / national sporting body monitoring your training?

- ◯ Every month (1)
- ◯ Every 2 weeks (2)
- ◯ Every week (3)
- ◯ Every couple of days (4)
- ◯ Every day (5)
- ◯ Other (6) ____

Display This Question:

If Is your sporting club / state sporting body / national sporting body monitoring your training? = Yes

Q8b How is your sporting club / state sporting body / national sporting body monitoring your training?

- ▢ Face to face (in person) (1)
- ▢ Video (e.g. skype) (2)
- ▢ Athletic Training Applications (3)
- ▢ Phone (4)
- ▢ Email (5)

Q9 Have you contracted COVID-19?

- Yes (1)
- Unsure (2)
- No (3)

Q10 Have any of your friends or family contracted COVID-19?

- Yes (1)
- Unsure (2)
- No (3)

Q11 Has anyone from your sporting club contracted COVID-19?

- Yes (1)
- Unsure (2)
- No (3)

Display This Question:

If Have you contracted COVID-19? = Yes

Q9a What was your experience having COVID-19?

- I became seriously ill (requiring hospitalization) (1)
- I became seriously ill (stayed at home) (2)
- I became moderately ill (3)
- I became slightly ill (4)
- No symptoms (5)

Page Break

Q12 How concerned are you about each of the following:

	Not concerned (1)	Slightly concerned (2)	Very concerned (3)	Extremely concerned (4)
Personally catching COVID-19 (1)	○	○	○	○
My friends or family members catching COVID-19 (2)	○	○	○	○
Losing personal income because of COVID-19 (3)	○	○	○	○
The ability of my sporting club (e.g. Toowoomba Tennis Club) to survive due to COVID-19 (10)	○	○	○	○
The ability of my state sporting body (e.g. Tennis Queensland) to survive due to COVID-19 (11)	○	○	○	○
The ability of my national sporting body (e.g. Tennis Australia) to survive due to COVID-19 (18)	○	○	○	○

Page Break

Q13 I think the response of my national sporting body to the COVID-19 outbreak has been:

▼ Very good (1) ... Very poor (5)

Q14 I think the response of my state sporting body to the COVID-19 outbreak has been:

▼ Very good (1) ... Very poor (5)

Q15 I think the response of my sporting club to the COVID-19 outbreak has been:

▼ Very good (1) ... Very poor (5)

End of Block: Impact

Start of Block: BEEPS

Q1 Over the past month I have generally been feeling physically:

	Not at all (1)	A little bit (2)	Quite a bit (3)	A lot (4)	Extremely (5)
Healthy (4)	○	○	○	○	○
Lethargic (5)	○	○	○	○	○
Strong (6)	○	○	○	○	○
Unfit (7)	○	○	○	○	○
Energetic (8)	○	○	○	○	○
Weak (9)	○	○	○	○	○

End of Block: BEEPS

Start of Block: BEES

Q1 Over the past month I have generally been feeling emotionally:

	Not at all (1)	A little bit (2)	Quite a bit (3)	A lot (4)	Extremely (5)
Happy (4)	○	○	○	○	○
Worried (5)	○	○	○	○	○
Calm (6)	○	○	○	○	○
Sad (7)	○	○	○	○	○
Confident (8)	○	○	○	○	○
Afraid (9)	○	○	○	○	○

End of Block: BEES

Start of Block: Brief Coping Scale

Q1 The following questions ask how you have sought to cope with COVID-19 in your life. Read the statements and indicate how much you have been using each coping style.

	I haven't been doing this at all (1)	I've been doing this a little bit (2)	I've been doing this a medium amount (3)	I've been doing this a lot (4)
I've been turning to work or other activities to take my mind off things. (1)	○	○	○	○
I've been concentrating my efforts on doing something about the situation I'm in. (2)	○	○	○	○
I've been saying to myself "this isn't real". (3)	○	○	○	○
I've been using alcohol or other drugs to make myself feel better. (4)	○	○	○	○
I've been getting emotional support from others. (5)	○	○	○	○
I've been giving up trying to deal with it. (6)	○	○	○	○
I've been taking action to try to make the situation better. (7)	○	○	○	○

Q2 Continued.

	I haven't been doing this at all (1)	I've been doing this a little bit (2)	I've been doing this a medium amount (3)	I've been doing this a lot (4)
I've been refusing to believe that is has happened. (8)	○	○	○	○
I've been saying things to let my unpleasant feelings escape. (9)	○	○	○	○
I've been getting help and advice from other people. (10)	○	○	○	○
I've been using alcohol or other drugs to help me get through it. (11)	○	○	○	○
I've been trying to see it in a different light, to make it seem more positive. (12)	○	○	○	○
Ive been criticizing myself. (13)	○	○	○	○
I've been trying to come up with a strategy about what to do. (14)	○	○	○	○

Page Break

Q3 Continued.

	I haven't been doing this at all (1)	I've been doing this a little bit (2)	I've been doing this a medium amount (3)	I've been doing this a lot (4)
I've been getting comfort and understanding from someone. (15)	○	○	○	○
I've been giving up the attempt to cope. (16)	○	○	○	○
I've been looking for something good in what is happening. (17)	○	○	○	○
I've been making jokes about it. (18)	○	○	○	○
I've been doing something to think about it less, such as watching movies or TV, reading, daydreaming, sleeping, shopping or working. (19)	○	○	○	○
I've been accepting the reality of the fact that it has happened. (20)	○	○	○	○
I've been expressing my negative feelings. (21)	○	○	○	○

Q4 Continued.

	I haven't been doing this at all (1)	I've been doing this a little bit (2)	I've been doing this a medium amount (3)	I've been doing this a lot (4)
I've been trying to find comfort in my religion or spiritual beliefs. (22)	○	○	○	○
I've been trying to get advice or help from other people about what to do. (23)	○	○	○	○
I've been learning to live with it. (24)	○	○	○	○
I've been thinking hard about what steps to take. (25)	○	○	○	○
I've been blaming myself for things that happened. (26)	○	○	○	○
I've been praying or meditating. (27)	○	○	○	○
I've been making fun of the situation. (28)	○	○	○	○

End of Block: Brief Coping Scale

Start of Block: Final Comments

Q1

If you have anything more to add about how you are currently feeling because of COVID-19 or the impacts it has had on you, please write some comments in the space below.

____

Q76 If you have anything more to add about the strategies you are using to cope with the COVID-19 situation, please write some comments in the space below.

Mental health resources This survey has included questions that ask about the impact of COVID-19 pandemic on your livelihood and physical and emotional well-being. If completing this survey has made you feel like you would like to talk to someone about any negative feelings that you might be experiencing, please be aware there are options available. In Australia, see links below to web pages which all list potential services (*If looking at any of these links please right-click on them and 'open in a new window', or in a new tab, so that the current page stays open). Government mental health web page: https://www.mentalhealth.gov/get-help LifeLine: https://www.lifeline.org.au/get-help/get-help-home Find a psychologist via Australian Psychological Society: https://www.psychology.org.au/Find-a-Psychologist Find a registered counsellor via the Australian Counselling Association: https://www.theaca.net.au/find-registered-counsellor.phpCOVID-19 support resource: https://headtohealth.gov.au/covid-19-support/covid-19

Thank you for taking the time to complete this survey. To finish the survey please click the 'next' button to lodge your responses.

End of Block: Final Comments
