# Supplementary material for: A Survey to Evaluate the Association of COVID-19 Restrictions on Perceived Mood and Coping in Australian Community Level Athletes
Source: Front Sports Act Living. 2021 Mar 22;3:624267. doi: 10.3389/fspor.2021.624267 (PMC8021018; doi:10.3389/fspor.2021.624267)
Supplement: Supplementary file 2 [file Table_1.docx]

| **Supplementary Table 1. Sporting classification** | | |
| --- | --- | --- |
| **Individual** | **Team** | **Other / Unknown** |
|  | Australian Football |  |
| Artistic Swimming | Basketball |  |
| Badminton | Cricket | * |
|  | Curling |  |
| Canoe Sprint | Hockey |  |
| Climbing | Netball |  |
| Gym / Weightlifting | Rugby Union |  |
| Cycling | Soccer |  |
| Diving | Ultimate Frisbee |  |
| Equestrian | Water Polo |  |
| Fencing |  |  |
| Golf |  |  |
|  |  |  |
| Lawn Bowls / Bowls |  |  |
| Rock Climbing |  |  |
| Running |  |  |
| Swimming |  |  |
| Table Tennis |  |  |
| Tenpin Bowling |  |  |
| Tennis |  |  |
| Track and Field |  |  |
| Triathlon |  |  |
| Yoga |  |  |
| Zumba |  |  |

** Other sport cannot be disclosed due to risk of identifying the respondent (n=1)*
